# A New Phenylpyrazoleanilide, Y-320, Inhibits Interleukin 17 Production and Ameliorates Collagen-Induced Arthritis in Mice and Cynomolgus Monkeys

**DOI:** 10.3390/ph7010001

**Published:** 2013-12-23

**Authors:** Hiroyuki Ushio, Seigo Ishibuchi, Koichi Oshita, Noriyasu Seki, Hirotoshi Kataoka, Kunio Sugahara, Kunitomo Adachi, Kenji Chiba

**Affiliations:** 1 Process Chemistry Research Laboratories, CMC Division, Mitsubishi Tanabe Pharma Corporation, 3-16-89, Kashima, Yodogawa-ku, Osaka-shi 532-8505, Japan; E-Mail: Ushio.Hiroyuki@mk.mt-pharma.co.jp; 2 Medicinal Chemistry Research Laboratories I, Research Division, Mitsubishi Tanabe Pharma Corporation, 1000, Kamoshida-cho, Aoba-ku, Yokohama, Kanagawa 227-0033, Japan; E-Mails: Ishibuchi.Seigo@mm.mt-pharma.co.jp (S.I.); Adachi.Kunitomo@mc.mt-pharma.co.jp (K.A.); 3 Pharmacology Research Laboratories I, Research Division, Mitsubishi Tanabe Pharma Corporation, 1000, Kamoshida-cho, Aoba-ku, Yokohama, Kanagawa 227-0033, Japan; E-Mails: Oshita.Koichi@mc.mt-pharma.co.jp (K.O.); Seki.Noriyasu@mb.mt-pharma.co.jp (N.S.); Kataoka.Hirotoshi@mk.mt-pharma.co.jp (H.K.); Sugahara.Kunio@me.mt-pharma.co.jp (K.S.); 4 Advanced Medical Research Laboratories, Research Division, Mitsubishi Tanabe Pharma Corporation, 1000, Kamoshida-cho, Aoba-ku, Yokohama, Kanagawa 227-0033, Japan

**Keywords:** interleukin 15 (IL-15), interleukin 17 (IL-17), phenylpyrazoleanilide, Y-320, immunomodulator, collagen-induced arthritis (CIA), rheumatoid arthritis (RA)

## Abstract

Interleukin (IL)-15 and IL-17 are thought to play an important role in the pathogenesis of rheumatoid arthritis (RA) because both pro-inflammatory cytokines are found in synovial fluid of RA patients. In this study, we examined the pharmacological profiles of Y-320, a new phenylpyrazoleanilide immunomodulator. Y-320 inhibited IL-17 production by CD4 T cells stimulated with IL-15 with IC_50_ values of 20 to 60 nM. Oral administration of Y-320 (0.3 to 3 mg/kg) significantly inhibited the development and progression of arthritis and joint destruction with reduction of IL-17 mRNA expression in arthritic joints of type II collagen-induced arthritis (CIA) in DBA/1J mice. Y-320 in combination with anti-murine tumor necrosis factor-α monoclonal antibody showed a synergistic effect on mouse CIA. Moreover, therapeutic treatment with Y-320 (0.3 and 1 mg/kg orally) ameliorated CIA in cynomolgus monkeys. Our results suggest that Y-320, an orally active inhibitor for IL-17 production, provides a useful therapy for RA.

## 1. Introduction

Rheumatoid arthritis (RA) is a chronic autoimmune disease in which imbalances in pro- and anti-inflammatory cytokines promote induction of autoimmunity, inflammation and joint destruction [1]. Interleukin (IL)-15 is a pro-inflammatory cytokine associated with several autoimmune diseases, particularly rheumatoid arthritis [2,3]. IL-15 has biological activities similar to IL-2 because IL-15 receptor (IL-15R) complex consists of CD125 (IL-15Rα) specific for IL-15, CD122 (IL-2Rβ), and CD132 (IL-2Rγ) [4,5]. It has been reported that IL-15 is produced excessively in the joints of rheumatoid arthritis (RA) patients and induces recruitment of T cells to arthritic joints and activates T cells to induce tumor necrosis factor (TNF)-α production [6,7,8]. In type II collagen-induced arthritis (CIA) in DBA/1J mice, the treatment with soluble mouse IL-15Rα or antagonist mutant of murine IL-15/Fc fusion protein inhibits development of CIA, joint destruction, and production of anti-type II collagen antibodies [9,10]. Furthermore, it has been demonstrated that the treatment with a monoclonal antibody (mAb) against human IL-15 (HuMax-IL15) ameliorates symptoms of active RA patents [11]. Recently, it has been reported IL-15 can induce IL-17 production by CD4 T cells [12].

IL-17 is a pro-inflammatory cytokine produced by various types of cells including CD4 T cells which are categorized as a new subset called Th17 cells [12,13,14,15,16,17,18]. IL-17 induces the production of IL-6, IL-8, CC chemokine ligand 2 (CCL2), granulocyte-macrophage colony-stimulating factor, matrix metalloproteinases, and prostaglandin E_2_ in various types of cells including macrophages, fibroblast cells and synovial cells [19]. It has been documented that CIA is significantly inhibited in IL-17 deficient mice or by treatment with anti-IL-17 mAb [20,21,22]. Furthermore, high levels of IL-17 as well as IL-15 were detected in synovial fluid of RA patients [8]. Based on these findings, IL-15 and IL-17 are thought to play an important role in the pathogenesis of RA.

**Figure 1:**
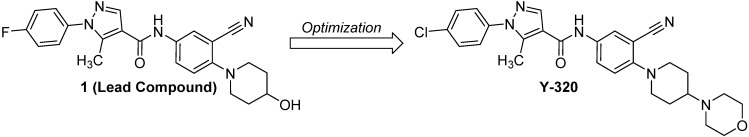
The structures of the lead compound **1** and Y-320.

In the research for a new orally active immunomodulator, we found a phenylpyrazoleanilide with a 4-hydroxypiperidine group (compound **1**) which inhibits proliferation of murine T cells induced by phorbol-12-myristate-13 acetate/calcium ionophore (A23187) and that of CTLL-2 cells induced by IL-15 [23]. Further chemical modification and optimization of **1** as a lead compound, we discovered a new immunomodulating compound, 1-(4-chlorophenyl)-*N*-[3-cyano-4-(4-morpholinopiperidin-1-yl)-phenyl]-5-methylpyrazole-4-carboxamide (Y-320), which potently inhibits IL-15-induced murine T cell proliferation and ameliorates CIA in DBA/1J mice (Figure 1) [24]. In the present study, we demonstrate that Y-320 inhibits IL-17 production by CD4 T cells stimulated with IL-15 and shows therapeutic effects on CIA in mice and cynomolgus monkeys.

## 2. Experimental Section

### 2.1. Chemistry

**Scheme 1:**
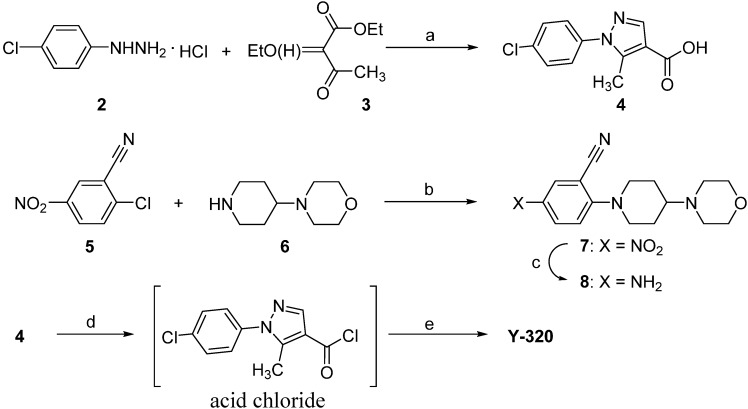
A convergent synthetic route for Y-320.

Melting points were determined in open capillary tubes with a Buchi 530 apparatus. ^1^H-NMR spectra were recorded on JEOL a JEOL GSX-400 (400 MHz) spectrometer (Tokyo, Japan), using tetramethylsilane as internal standard. Elemental analysis was performed on a Yanaco Group CORDER MT-6. Mass spectra (MS) were recorded on a JEOL JMS-700 instrument. IR spectra were recorded on JIR-6500W instrument. All chemicals and solvents were purchased from Sigma-Aldrich (Tokyo, Japan) and used without further purification. The synthetic scheme for Y-320 is shown in Scheme 1. 4-Morpholinopiperidine (**6**) and an equimolar of triethylamine were added to a solution of 2-chloro-5-nitrobenzonitrile (**5**) in acetonitrile to give an adduct **7**. The adduct **7** was converted via a reduction reaction with Fe (powder) and NH_4_Cl to give an aniline **8**. At the final step, 1-(4-chlorophenyl)-5-methylpyrazole-4-carboxylic acid (**4**) was treated with SOCl_2_ to give the corresponding acid chloride, which was coupled with **8** to afford Y-320. Compound **4** is now commercially available; however, it was derived via a ring formation reaction of phenylhydrazine HCl **2** and an ethyl acetoacetate derivative **3**.

Reagents and conditions: (**a**) i: NaHCO_3_/aq. EtOH, reflux; ii: aq. NaOH; (**b**) Et_3_N/CH_3_CN, reflux; (**c**) Fe, NH_4_Cl/ aq. EtOH, reflux; (**d**) SOCl_2_/toluene; (**e**) aniline **8**/pyridine, 40 °C.

### 2.2. The General Procedure for the Synthesis of Y-320

*1-(4-Chlorophenyl)-5-methylpyrazole-4-carboxylic acid* (**4**). 4-Chlorophenylhydrazine monohydro-chloride (**2**, 300 g), sodium hydrogen carbonate (140 g), and ethyl 2-ethyoxymethyleneacetoacetate (**3**, 312 g) prepared according to the literature [25] were added to 90% aqueous ethanol (1.2 L) and the reaction mixture was stirred for 3 h at a reflux temperature. To the reaction solution, sodium hydroxide (80 g) and water (600 mL) were added and the reaction mixture was stirred for 2 h at a reflux temperature and then concentrated *in vacuo*. The organic layer was separated with toluene and the aqueous layer was neutralized with conc. HCl to afford a precipitate, which was recrystallized from ethyl acetate to give compound **4** as a light yellow crystalline powder. Yield: 63% (for 2 steps). Mp: 193 °C. ^1^H-NMR (400 MHz, DMSO-*d_6_*) δ: 2.52 (3H, s), 7.60 (4H, dd, *J* = 3.3, 6.6 Hz), 7.98 (1H, s), 12.5 (1H, br). EIMS *m*/*z* 236 (M+): 219. Anal. Calcd. for C_11_H_9_ClN_2_O_2_: C, 55.83; H, 3.83; N, 11.84. Found: C, 55.59; H, 3.85; N, 11.89.

*2-(4-Morpholinopiperidin-1-yl)-5-nitrobenzonitrile* (**7**). To a solution of 2-chloro-5-nitrobenzonitrile (**5**) (38.6 g) in acetonitrile (115 mL), 4-morpholinopiperidine (**6**) (33.4 g) and triethylamine (43.2 g) were added and the reaction solution was stirred for 1 h at a reflux temperature. The reaction mixture was quenched with a saturated NaHCO_3_ solution and the organic layer was extracted with CHCl_3_ and was concentrated *in vacuo* to give compound **6** as a yellow solid (64.9 g). Yield: 97%. Mp: 103 °C. ^1^H-NMR (DMSO-*d_6_*) δ: 1.51–1.59 (2H, m), 1.95 (2H, d, *J* = 11.3 Hz), 2.25–2.61 (4H, m), 3.13 (2H, dd, *J* = 11.3, 11.7 Hz), 3.25–3.30 (1H, m), 3.59 (4H, brs), 3.98 (2H, d, *J* = 11.7 Hz), 7.25 (1H, d, *J* = 9.7 Hz), 8.26 (1H, dd, *J* = 3.0, 9.7 Hz), 8.50 (1H, d, *J* = 3.0 Hz). EIMS *m*/*z*: 316 (M^+^). Anal. Calcd. for C_16_H_20_N_4_O_3_: C, 60.75; H, 6.37; N, 17.71. Found: C, 60.58; H, 6.36; N, 17.54.

*5-Amino-2-(4-morpholinopiperidin-1-yl)benzonitrile* (**8**). To a mixed solution of compound **7** (64.9 g) and ammonium chloride (7.4 g) in water (170 mL) and EtOH (520 mL), iron powder (40 g) was added gradually under vigorous stirring and the reaction mixture was stirred for further 3 h. The reaction mixture was filtrated with celite and the filtrate was concentrated *in vacuo*. The residue was recrystallized from aqueous MeOH to yield compound **8** as an orange powder (50.2 g). Yield: 79%. Mp: 174 °C. EIMS *m*/*z*: 286 (M+). ^1^H-NMR (DMSO-*d_6_*) δ: 1.50 (1H, dd, *J* = 3.4, 11.7 Hz), 1.56 (1H, dd, *J* = 3.4, 12.2 Hz), 1.85 (2H, d, *J* = 11.2 Hz), 2.18–2.24 (1H, m), 2.47–2.50 (4H, m), 2.60 (1H, d, *J* = 10.2 Hz), 2.63 (1H, d, *J* = 11.7 Hz), 3.35–3.56 (4H, m), 5.17 (2H, brs), 6.78–6.80 (2H, m), 6.94 (1H, d, *J* = 9.7 Hz). Anal. Calcd. for C_16_H_22_N_4_O·1/10H_2_O: C, 66.69; H, 7.69; N, 19.44. Found: C, 66.55; H, 7.68; N, 19.14.

*1-(4-Chlorophenyl)-N-[3-cyano-4-(4-morphlinopiperidin-1-yl)phenyl]-5-methylpyrazole-4-carbox-amide* (Y-320). To a suspension of compound **4** (21.2 g) in toluene, SOCl_2_ (7.0 mL) was dropped and the reaction mixture was stirred at 60 °C for 3 h. The solvent was concentrated *in vacuo* to give the corresponding acid chloride. To this residue, a solution of compound **8** (23 g) in pyridine (200 mL) was added and the reaction solution was stirred at 40 °C for 3 h. An aqueous K_2_CO_3_ solution was poured into the reaction mixture to give a yellow precipitate, which was separated by filtration and purified by recrystallization from an acetone-EtOH (1:1) mixed solution to give Y-320 (37 g) as a light yellow powder. Yield: 92%. Mp: 260 °C/dec. ^1^H-NMR (DMSO-*d_6_*) δ: 1.58 (2H, dddd, *J* = 3.7, 10.8, 11.0, 11.2 Hz), 1.90 (2H, d, *J* = 10.8 Hz), 2.29 (1H, tt, *J* = 3.7, 11,2 Hz), 2.47–2.51 (4H, m), 2.56 (3H, s), 2.77 (2H, dd, *J* = 11.0, 12.2 Hz), 3.47 (2H, d, *J* = 12.2 Hz), 3.59 (4H, m), 7.17 (1H, d, *J* = 8.8 Hz), 7.59 (2H, d, *J* = 9.3 Hz), 7.63 (2H, d, *J* = 9.3 Hz), 7.85 (1H, dd, *J* = 2.4, 8.8 Hz), 8.06 (1H, d, *J* = 2.4 Hz), 8.30 (1H, s), 9.94 (1H, brs). IR (KBr) ν: 3373, 2958, 2924, 2852, 2828, 2225, 1668, 1587, 1554, 1525, 1500, 1309, 879, 839 cm^−1^. EIMS *m*/*z*: 505 (M+). Anal. Calcd. for C_27_H_29_ClN_6_O_2_: C, 64.21; H, 5.79; N, 16.64. Found: C, 64.07; H, 5.76; N, 16.57.

### 2.3. Animals

Inbred strains of male DBA/1J mice were purchased from Charles River Japan (Atsugi, Kanagawa, Japan) and were used at 6 to 8 weeks of age. Female cynomolgus monkeys were used at 3 to 5 years old of ages in the experiments (Sin-Nippon Kagaku Co., Kagoshima, Japan). All animal experiments were performed under an experimental protocol approved the ethics review committee for animal experimentation of Research Division, Mitsubishi Tanabe Pharma Corporation.

### 2.4. IL-17 Production by CD4 T Cells Stimulated with IL-15

Splenic or lymph node CD4 T cells (>95%) from DBA/1J mice were purified by passing through mouse CD4 subset enrichment columns (R&D Systems, Minneapolis, MN, USA) and cultured in RPMI 1640 medium (Sigma-Aldrich, St Louis, MO, USA) supplemented with 10% fetal calf serum (FCS, Gibco BRL, Grand Island, NY, USA), 10 mM HEPES, 100 U/mL penicillin G, 60 µg/mL kanamycin sulfonate, and 50 µM 2-mercaptoethanol (10%FCS-RPMI 1640 medium). Murine CD4 T cells (5 × 10^5^ cells/well) were stimulated with 100 ng/mL of recombinant mouse (rm)-IL-15 (Research Diagnostics Inc., Flanders, NJ, USA), 1,000 ng/mL of rm-CXC-chemokine ligand 12 (CXCL12, R&D Systems), and hamster anti-mouse CD3ε monoclonal antibody (mAb) (clone: 145-2C11, plate-precoated at 1 µg/mL, BD Biosciences, San Diego, CA, USA) in 10% FCS-RPMI1640 medium in the presence or absence of Y-320 and cultured for 48 h at 37 °C in 5% CO_2_. In other experiments, human CD4 T cells (5 × 10^5^ cells/well) prepared from peripheral blood of healthy volunteers were stimulated with recombinant human (rh) IL-15 (Genzyme/Techne, Cambridge, MA, USA) for 48 h. After the culture, the amounts of IL-17 in the culture supernatants were determined by enzyme-linked immunosorbent assay (ELISA) kit for mouse or human IL-17 (Genzyme/Techne). The amounts of interferon (IFN)-γ and TNF-α were also determined by ELISA for mouse or human IFN-γ and TNF-α (Genzyme/Techne).

### 2.5. Generation of Th1 and Th17 Cells *in Vitro*

Murine splenic CD4 T cells (10^6^ cells) were stimulated with anti-CD3 mAb (10 µg/mL) and anti-CD28 mAb (1 µg/mL, clone: 37.51, eBiosciences, San Diego, CA, USA) in 10%FCS-RPMI 1640 medium for 48 h under Th17 condition: rm-IL-6 (20 ng/mL, Peprotech Inc., Rocky Hill, NJ, USA), rm-transforming growth factor (TGF)-β1 (10 ng/mL, Peprotech), anti-IFN-γ mAb (10 µg/mL, clone: RMMG-1, Biosourse, Camarillo, CA, USA), and rat anti-mouse IL-4 mAb (20 µg/mL, clone: 11B11, Biosource) [26,27]. Then the cells were added rm-IL-23 (5 ng/mL, R&D Systems) and cultured for additional 96 h. Proportions of Th1/Th17 cells were analyzed by intracellular cytokine staining using anti-mouse IFN-γ and IL-17 mAbs according to the method as described previously [28,29]. The recovered cells (2.5 × 10^4^ cells) were re-stimulated with anti-CD3 mAb plus anti-CD28 mAb for 24 h and IL-17 in the supernatants were determined by ELISA.

### 2.6. Phosphorylation of Janus Kinase (JAK) 1 and JAK3 by IL-15

Mouse splenic CD4 T cells (5 × 10^6^ cells) were stimulated with rm-IL-15, rm-CXCL12 and anti-mouse CD3 mAb as described above in 10% FCS-RPMI1640 medium in the presence or absence of Y-320 and cultured for 48 h. After the culture, the cells were lysed with 50 mM Tris-HCl buffer (pH 7.5) containing 50 mM β-glycerophosphate, 1 mM Na_3_VO_4_, 1 mM phenylmethylsulfonyl fluoride and 0.1% Triton X-100. The cell lysates were immunoprecipitated with rabbit anti-JAK1 polyclonal antibody (pAb) (Santa Cruz Biotechnology, Inc., Santa Cruz, CA, USA) or rabbit anti-JAK3 antiserum (Upstate Biotechnology, Lake Placid, NY, USA) with protein A-agarose for 2 h at 4 °C [30,31]. Then the immunoprecipitates were subjected to SDS-PAGE. After transfer to PVDF membrane filters (Amersham Pharmacia Biotech, Uppsala, Sweden), immunoblotting of phosphorylated-JAK1 (p-JAK1) and p-JAK3 was performed with enhanced chemiluminescemce using anti-mouse phosphotyrosine mAb (clone: 4G10, Upstate Biotechnology), horse radish peroxidase (HRP)-conjugated goat anti-mouse immunoglobulin G (IgG) (Santa Cruz Biotechnology, Inc.). Immunoblotting of JAK1 or JAK3 was performed using anti-JAK1 pAb or rabbit anti-JAK3 antiserum, respectively.

### 2.7. CIA in DBA/1J Mice

CIA was induced by immunization with the 200 µg bovine type II collagen (Collagen Research Center) and Freund’s complete adjuvant (Sigma-Aldrich) by injecting subcutaneously into the tail base of DBA/1J mice, followed by a booster injection after 21 days [32,33,34]. In other experiments, chronic-progressing CIA was induced by a single immunization with 200 µg bovine type II collagen and Freund’s complete adjuvant. Y-320 was suspended in a 0.5% hydroxypropylmethylcellulose (HPMC, Shinetsu Chemical Co., Tokyo, Japan) solution and administered orally. Anti-murine TNF-α mAb (clone: TN3-19.12, BD Biosciences) [35,36,37] diluted with saline was administered intravenously. Arthritis scores were evaluated according to the following criteria. Score 0, no change; score 1, edema at one joint; score 2, edema at two or more joints, or mild edema throughout the limbs; score 3, severe edema throughout the limbs; score 4, severe edema throughout the limbs and ankylosis [32,33,34]. The total paw thickness in four limbs was measured by using a digital caliper. On the next day of final administration, soft X-ray photos of the limbs were taken and joint destruction was evaluated under microscope according to the following criteria. Score 0, no change, score 1; one or more joint destructions in a finger; score 2, the morphological change of joint area in a finger. The titers of anti-type II collagen IgG1 in the plasma were determined by ELISA using bovine type II collagen as an antigen and HRP-conjugated anti-mouse IgG1 as a secondary antibody. In some experiments, mouse ankle joints were decalcified in 10% ethylenediaminetetraacetic acid, dehydrated in ethanol, and embedded in paraffin. Histological examinations of 6 µm sections were performed by hematoxylin and eosin (H&E) staining and Safranin O staining.

### 2.8. CIA in Cynomolgus Monkeys

CIA was induced by immunization with the 1 mg bovine type II collagen (Collagen Research Center, Tokyo, Japan) and Freund’s complete adjuvant by intradermal injection into the back skin of cynomolgus monkeys three times with 3-week intervals [38,39]. At 3-week after the final immunization, CIA-established monkeys were selected and Y-320 suspended in 0.5% HPMC solution was administered orally. The paw thickness in each limb was measured by using a digital caliper and total paw thickness was calculated by the sum of those in four limbs.

### 2.9. Real Time Polymerase Chain Reaction

Arthritic ankle joints of CIA mice were removed and the total RNA was extracted using TRIZOL (Invitrogen, Carlsbad, CA, USA) and a tow-step quantitative revers transcription-polymerase chain reaction (RT-PCR) was performed to determine various molecules mRNA expression using a relative standard curve method with cellular housekeeping enzyme (glyceraldehyde 3-phosphate dehydrogenase: GAPDH) as normalization control. Complementary DNA was synthesized with TaqMan Reverse Transcription Reagents (Applied Biosystems, Foster City, CA, USA) using random hexamers and 0.5 µg of total RNA. Complementary DNA was amplified with various molecules TaqMan probe (6-carboxyfluorecein label)/primer, cellular housekeeping enzyme TaqMan probe (VIC label)/primer, and TaqMan Universal PCR Master Mix in an ABI PRISM 7900 Sequence Detection System (Applied Biosystems). For analyses of mRNA expressions in mouse knee joints, following TaqMan probes/primers were used: IL-17 (Mm00439619_m1), IL-6 (Mm00446190_m1), TNF-α (Mm00443258_m1), and CCL2 (Mm00441242_m1). GAPDH TaqMan probe/primer was used as normalization control. For quantification, standard curves were generated for various molecules using serially diluted cDNA samples from the joints of CIA mice. The level of mRNA in every sample was normalized by calculating the ratio of each target molecule/GAPDH level.

### 2.10. Statistical Analysis

Results were expressed as the mean ± standard error of the mean (SEM). Statistical differences were calculated by Student’s *t*-test or Dunnett’s multiple comparison test. Differences between groups were considered significant when *p* < 0.05.

## 3. Results and Discussion

### 3.1. Y-320 Inhibits IL-17 Production by Murine and Human CD4 T Cells Stimulated with IL-15

RA is characterized by accumulation of CD4^+^ CD45RO^+^ memory T cells in inflamed synovium. As reported previously, high levels of IL-17 as well as IL-15 were detected in synovial fluid of patients with RA but not osteoarthritis [8]. Moreover, it has been reported that the frequency of CXC chemokine receptor 4 (CXCR4) expressing memory CD4 T cells is significantly elevated in synovial tissues in RA patients and CXCR4 expression is induced by IL-15 [40]. CXCL12, the ligand of CXCR4 is a chemoattractant for T cells, B cells, monocytes, and neutrophils and is expressed in RA synovium. In addition, CXCL12 co-stimulates proliferation of CD4 T cells by anti-CD3-activation [41]. Based on these findings, it is presumed that IL-15 and CXCL12 play an important role in IL-17 production by memory CD4 T cells in RA synovium.

**Figure 2:**
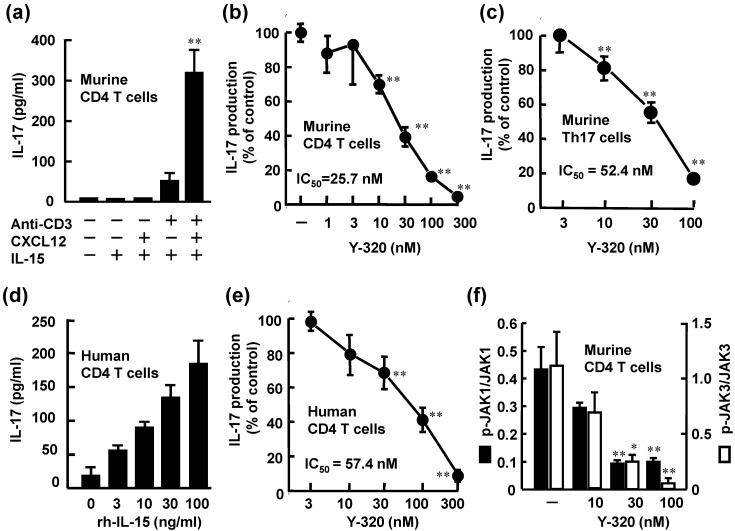
Y-320 inhibits IL-17 production by murine and human CD4 T cells stimulated with IL-15. (**a**) rm-IL-15 (100 ng/mL) with rm-CXCL12 and anti-CD3 mAb induces IL-17 production by murine CD4 T cells; (**b**) Y-320 inhibits IL-17 production by murine CD4 T cells stimulated with IL-15/CXCL12/anti-CD3 mAb; (**c**) Y-320 inhibits IL-17 production by murine Th17 cells; (**d**) rh-IL-15 induces IL-17 production by human CD4 T cells; (**e**) Y-320 inhibits IL-17 production by human CD4 T cells stimulated with IL-15 (100 ng/mL); (**f**) Y-320 inhibits phosphorylation of JAK1/JAK3 in murine CD4 T cells stimulated with IL-15/CXCL12/anti-CD3 mAb. Results were expressed as the mean ± SEM of triplicated determination. * *p* < 0.05, ** *p* < 0.01 (Dunnett’s multiple comparison test).

In this study, we found that a significant level of IL-17 is produced by mouse CD4 T cells stimulated with IL-15, CXCL12 and, anti-CD3 mAb, though no detectable level of IL-17 was induced by IL-15 alone (Figure 2a). Under this condition, IL-17 was predominantly produced by CD62L^low^, CD44^high^ memory type CD4 T cells, but not naïve CD4 T cells (data not shown).

Y-320 at 10 nM or higher significantly inhibited IL-17 production of murine CD4 T cells stimulated with IL-15, CXCL12, and anti-CD3 mAb in a dose-dependent manner with IC_50_ values of 25.7 nM (Figure 2b). Under this experimental condition, significant levels of IFN-γ and TNF-α as well as IL-17 were produced by mouse CD4 T cells. Y-320 also inhibited the production of IFN-γ and TNF-α by mouse CD4 T cells stimulated with IL-15, CXCL12, and anti-CD3 mAb; however Y-320 showed 20- to 50-fold more potent inhibitory effect on IL-17 production than IFN-γ or TNF-α production (data not shown). In addition, our preliminary data suggest that Y-320 did not affect the production of IL-6 or TNF-α by mouse macrophages stimulated with lipopolysaccharide (data not shown). Based on these results, we focused on the effects of Y-320 on IL-17 production by mouse or human CD4 T cells.

As shown in Figure 2c, Y-320 inhibited IL-17 production by Th17 cells with IC_50_ value of 52.4 nM in a dose-dependent manner. On the other hand, this compound did not affect generation of Th17 cells from murine naïve CD4 T cells under the Th17 condition (in the presence of IL-6, TGF-β1, and IL-23) (data not shown). In human peripheral blood CD4 T cells, a significant level of IL-17 was produced by stimulation with rh-IL-15 (3 to 100 ng/mL). Y-320 (10 to 300 nM) significantly inhibited IL-17 production by human CD4 T cells stimulated with rh-IL-15 (100 ng/mL) with an IC_50_ value of 57.4 nM (Figure 2d). These results clearly indicate that Y-320 inhibits IL-17 production from murine and human CD4 T cells including Th17 cells and memory type CD4 T cells stimulated with IL-15.

It has been known that IL-15 binding to IL-15R complex induces phosphorylation of both JAK1 and JAK3 [29,30]. Y-320 at 30 nM or higher significantly inhibited the phosphorylation of JAK 1 and JAK3 with IC_50_ values of 15.7 and 13.5 nM, respectively (Figure 2f). On the other hand, Y-320 up to 300 nM did not affect the activities of more than 30 kinds of kinases including JAK1, JAK2, JAK3, p38 mitogen-activated protein kinase and protein kinase C (data not shown). These results suggest that the inhibition of JAK phosphorylation by Y-320 is not mediated by its direct inhibition of enzyme activity. Further studies are necessary to understand the molecular mechanism of action of Y-320.

### 3.2. Y-320 Ameliorates CIA in Mice with a Reduction of IL-17 mRNA in Arthritic Joints

**Figure 3:**
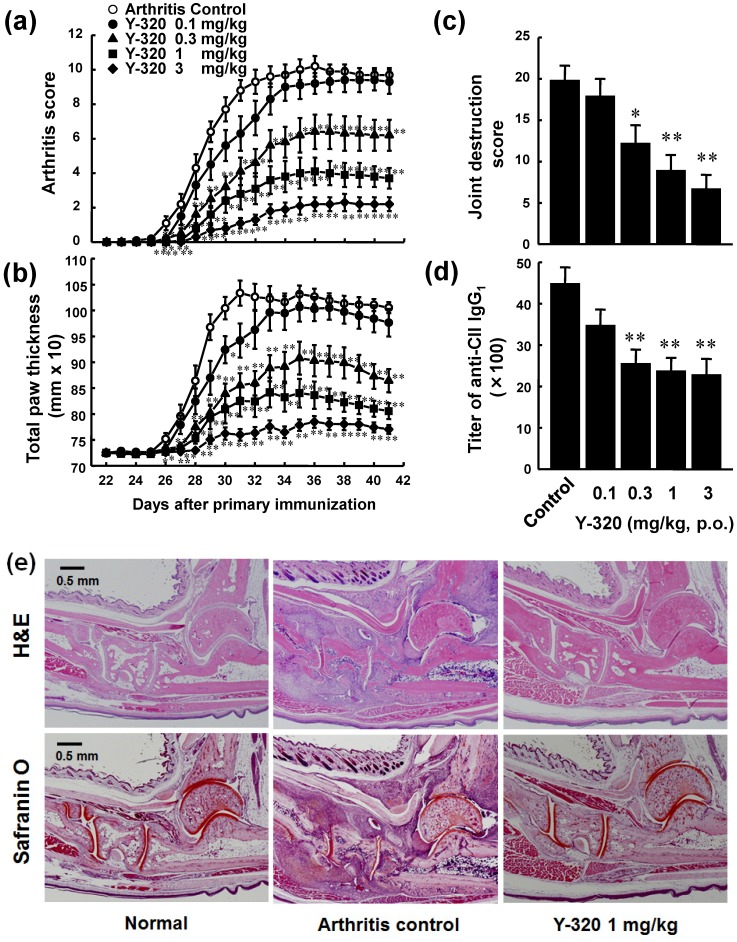
Prophylactic administration of Y-320 inhibits CIA in DBA/1J mice. Y-320 was administered orally to mice from the day of primary immunization for 6 weeks. (**a**) Arthritis score; (**b**) Joint swelling; (**c**) Joint destruction score; (**d**) Anti-type II collagen (CII) IgG1 in the plasma. Results were expressed as the mean ± SEM of 10 mice. * *p* < 0.05, ** *p* < 0.01 (Dunnett’s multiple comparison test); (**e**) H&E staining and Safranin O staining.

Because Y-320 effectively inhibits IL-17 production by mouse CD4 T cells *in vitro*, we examined therapeutic potential of Y-320 on CIA in DBA/1J mice. CIA was induced by immunization with type II collagen and Freund’s complete adjuvant on day 0 and 21. Y-320 was administered orally every day for 42 days from the day of primary immunization. In the control group, arthritis scores started to increase gradually from 3 days after the booster immunization, reaching a maximum level of 10.2 at 36 days after primary immunization and thereafter (Figure 3a). Prophylactic administration of Y-320 at 0.3 mg/kg or higher significantly inhibited the development of CIA and the increase in paw thickness in a dose-dependent manner (Figure 3b). Furthermore, Y-320 (0.3 mg/kg or higher) significantly inhibited joint destructions in a dose-dependent manner (Figure 3c) and reduced the titers of anti-type II collagen IgG1 in the plasma of CIA mice (Figure 3d). On the next day of final administration, we performed histological examinations of arthritic ankle joints in CIA mice. Figure 3e shows typical data of H&E staining and Safranin O staining of joints in normal mice, CIA mice (arthritis control), and CIA mice treated with Y-320 at 1 mg/kg for 6 weeks. We observed erosion of cartilage and bone and infiltration of inflammatory cells including lymphocytes and neutrophils in the joints of CIA mice. Our histological examinations revealed that treatment with Y-320 appeared to improve inflammation and damage in the arthritic ankle joints in CIA mice.

**Figure 4:**
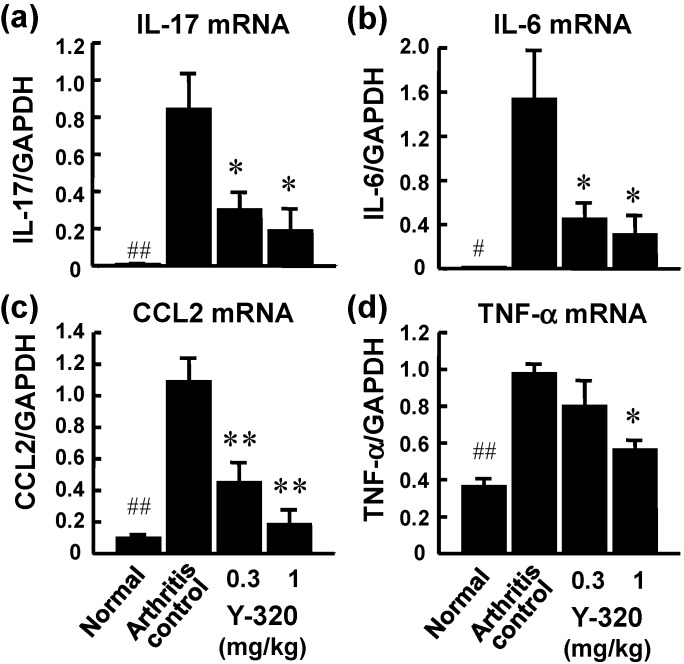
The mRNA expressions of pro-inflammatory cytokines in the arthritic joints of CIA mice treated with Y-320. Y-320 was administered orally to mice from the day of primary immunization for 6 weeks. (**a**) IL-17 mRNA; (**b**) IL-6 mRNA; (**c**) CCL2 mRNA; (**d**) TNF-α mRNA. Results were expressed as the mean ± SEM of 4 mice. * *p* < 0.05, ** *p* < 0.01 (Dunnett’s multiple comparison test). ^#^ *p* < 0.05, ^##^ *p* < 0.01 (Student’s *t*-test).

On the next day of final administration, mRNA expressions of pro-inflammatory cytokines in arthritic ankle joints were examined by real time RT-PCR. The mRNA levels of IL-17, IL-6, CCL2, and TNF-α were markedly elevated in the arthritic ankle joints in control CIA mice. Administration of Y-320 (0.3 and 1 mg/kg) resulted in a significant reduction of mRNA levels of IL-17, IL-6, and CCL2, (Figure 4a–c), whereas it showed a relatively weak reduction of TNF-α mRNA level (Figure 4d). Similarly, treatment with anti-mouse TNF-α mAb showed significant reduction of mRNA levels of IL-6 and CCL-2 but did no significant reduction of IL-17 mRNA level (data not shown). These results strongly suggest that Y-320 ameliorates CIA in mice by inhibiting IL-17 production by antigen-specific CD4 T cells and that mode of action of Y-320 is different from that of anti-TNF-α therapy.

### 3.3. Y-320 Shows Therapeutic Effects and a Synergistic Effect in Combination with Anti-TNF-α mAb on Chronic-Progressing CIA in Mice

**Figure 5:**
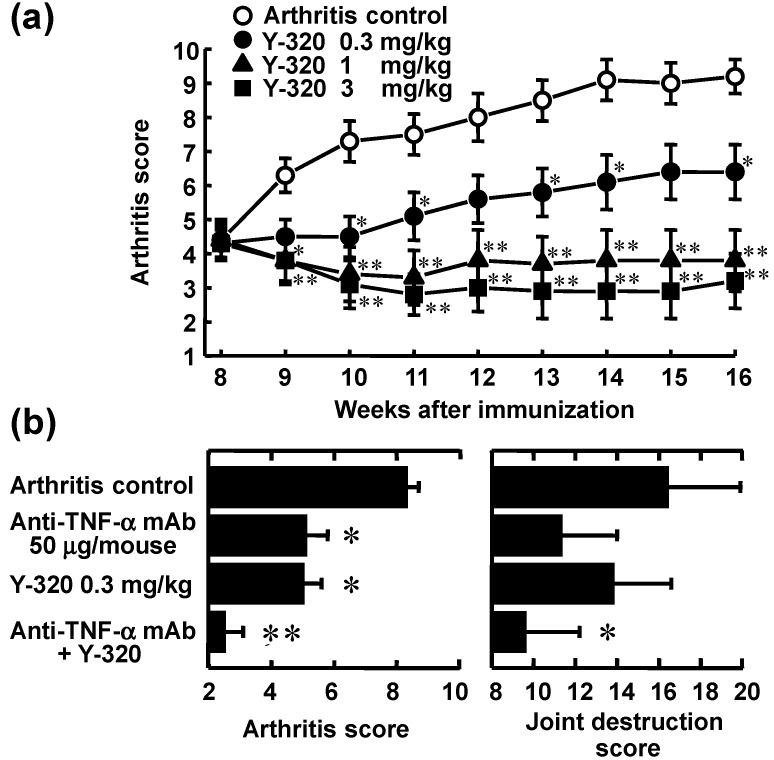
Therapeutic administration of Y-320 ameliorates chronic-progressing CIA in DBA/1J mice. (**a**) Y-320 was administered orally for 8 weeks; (**b**) Y-320 was administered orally for 4 weeks and anti-murine TNF-α mAb was given by a single intravenous injection. Results were expressed as the mean ± SEM of 12 mice. * *p* < 0.05, ** *p* < 0.01 [(a): Dunnett’s multiple comparison test; (b): Student’s *t*-test].

Next, we examined the therapeutic effect of Y-320 on chronic-progressing CIA induced by a single immunization with type II collagen and Freund’s complete adjuvant to DBA/1J mice. Eight weeks after the immunization, Y-320 was therapeutically administered to CIA-established mice for 8 weeks. In the control group, arthritis score increased gradually from 8 to 14 weeks, reaching a plateau mean value of approximately 9.0 at 14 weeks after the immunization and thereafter. The increase in arthritis score was significantly inhibited in the groups given 0.3 mg/kg or higher doses of Y-320. Especially, at 1 and 3 mg/kg, the increase in arthritis score was almost completely inhibited and the value of arthritis score at post-administration of Y-320 was maintained to the similar level of pre-administration. Similarly, the swelling of paws was significantly inhibited in the groups given 0.3 mg/kg or higher doses of Y-320, and the paw thickness in the groups given Y-320 at 1 and 3 mg/kg remained almost unchanged from the values showed pre-administration with the swelling being prevented almost completely. The joint destruction scores evaluated on the next day of final administration were significantly lower in the group given Y-320. These results indicate that Y-320 at oral doses of 0.3 to 3 mg/kg shows a significant therapeutic effect in chronic progressing CIA in DBA/1J mice (Figure 5a).

It has been reported that the progression of CIA in mice was markedly inhibited by treatment with anti-murine TNF-α mAb, TN3-19.12 [35,36,37]. In this study, we examined the concomitant effect of Y-320 with anti-murine TNF-α mAb, TN3-19.12 on chronic-progressing CIA in DBA/1J mice. The arthritis scores in the monotherapy group given Y-320 at 0.3 mg/kg orally or anti-TNF-α mAb 150 µg/mice intravenously were significantly lower as compared with control group. The arthritis scores and joint destruction scores in the concomitant groups given Y-320 with anti-TNF-α mAb were significantly lower when compared with each monotherapy group (Figure 5b). These results suggest that therapeutic administration of anti-TNF-α mAb in combination with Y-320 shows a synergistic effect on chronic-progressing CIA in mice.

### 3.4. Y-320 Shows Therapeutic Effects on CIA in Cynomolgus Monkeys

**Figure 6:**
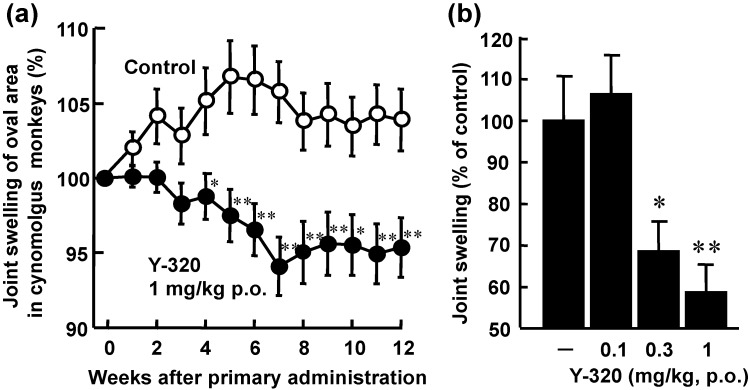
Therapeutic administration of Y-320 ameliorates CIA in cynomolgus monkeys. Y-320 was administered orally to monkeys from 9-week after primary immunization for 12 weeks. (**a**) Y-320 at 1 mg/kg showed a significant reduction of joint swelling; (**b**) Y-320 (0.3 and 1 mg/kg) showed a significant and dose-dependent inhibition of joint swelling. Results were expressed as the mean ± SEM of 12 monkeys. * *p* < 0.05, ** *p* < 0.01 [(a): Student’s *t*-test; (b): Dunnett’s multiple comparison test].

Because Y-320 had a relatively higher bioavailability in mice and monkeys (data not shown), we examined the therapeutic effects of Y-320 on CIA in cynomolgus monkeys. After immunization with type II collagen and Freund’s complete adjuvant, CIA-established monkeys were selected and used for therapeutic administration of Y-320. In vehicle-treated control groups, joint swelling was maintained during the administration period. Therapeutic administration of Y-320 at an oral dose of 1 mg/kg showed a significant reduction of joint swelling as compared with control group. Furthermore, therapeutic administration of Y-320 (0.3 and 1 mg/kg, orally for 12 weeks) showed a significant and dose-dependent inhibition of joint swelling in monkey CIA (Figure 6).

## 4. Conclusions

IL-15 and IL-17 are thought to play an important role in the pathogenesis of RA and related autoimmune diseases. In the research for a new orally active immunomodulator, we discovered Y-320, a phenylpyrazoleanilide which inhibits IL-15-induced proliferation of murine T cells. In this study, we found that Y-320 inhibits IL-17 production by IL-15-stimulated CD4 T cells, including Th17 cells and memory type CD4 T cells in mice and humans. Therapeutic administration of Y-320 improved arthritic symptoms, joint swelling, and joint destruction in mouse CIA and showed a synergistic effect in combination with anti-TNF-α mAb. Furthermore, Y-320 effectively ameliorated CIA in cynomolgus monkeys. Based on these findings, Y-320 that targets IL-15 and IL-17 could provide a new useful therapy for RA and related autoimmune diseases.
